# Simulation of Design Factors of a Clutch Pack for Power-Shift Transmission for an Agricultural Tractor

**DOI:** 10.3390/s20247293

**Published:** 2020-12-18

**Authors:** Md. Abu Ayub Siddique, Wan-Soo Kim, Yeon-Soo Kim, Seung-Yun Baek, Seung-Min Baek, Yong-Joo Kim, Seong-Un Park, Chang-Hyun Choi

**Affiliations:** 1 Department of Biosystems Machinery Engineering, Chungnam National University, Daejeon 34134, Korea; engg.ayub64@gmail.com (M.A.A.S.); wskim0726@gmail.com (W.-S.K.); kimtech612@gmail.com (Y.-S.K.); 2 Smart Agricultural Machinery R&D Group, Korea Institute of Industrial Technology (KITECH), Gimje 54325, Korea; 3 Department of Smart Agriculture Systems, Chungnam National University, Daejeon 34134, Korea; kelpie0037@gmail.com (S.-Y.B.); bsm1104@naver.com (S.-M.B.); 4 Reliability Test Team, TYM ICT Co. Ltd., Gongju 32530, Korea; 5 Department of Bio-Mechatronics Engineering, Sungkyunkwan University, Suwon 16419, Korea; choiauto@skku.edu

**Keywords:** tractor, powershift transmission, design factors, simulation model, clutch pack

## Abstract

The objective of this study is the simulation of the most affected design factors and variables of the clutch pack for the power-shift transmission (PST) of a tractor based measured data. The simulation model, the mathematical model of sliding velocity, a moment of inertia, and clutch engagement pressure of clutch pack were developed using the powertrain and configurations of the real PST tractor. In this study, the sensor fusion method was used to precisely measure the proportional valve pressure by test bench, which was applied to the simulation model. The clutch engagement times were found 1.20 s at all temperatures for determined factors. The engagement pressures have a significant difference at various temperatures (25 to 100 °C) of the hydraulic oils after the 1.20 s but the most affected factors were satisfied with the simulation conditions that ensure the clutch engagement on time. Finally, this sensor fusion method is believed to be helpful in realizing precision agriculture through minimization of power loss and maximum energy efficiency of tractors.

## 1. Introduction

Agricultural tractors have to deal with various working conditions providing large traction forces at low speed. Advanced technologies such as autonomous, artificial intelligence are applying in the agricultural tractors considering driving comfort, working efficiency, and precision farming [1]. To confirm the users’ flexibility, precise work, and maximum available power in all conditions during operations, several researchers and manufacturers are developing numerous powertrain system that has included the manual transmission (MT), automatic transmission (AT), dual-clutch transmission (DCT), continuously variable transmission (CVT), and power-shift transmission (PST) for agricultural tractors [2,3,4].

Recently, the PST is the most popular advanced precision technology for being comparatively easier and convenient to control and maintain the vehicle on-field for the users. The PST allows changing gear stages precisely on the run under load conditions of the vehicles [5]. There are two types of PST such as the partial and full PST. The partial PST deals with two or more speeds without a clutch, although it must have clutched to shift gears, and a full PST means the users can shift all gears without clutching [6]. Generally, the MT, which has the highest power transmission efficiency, is almost 96%. AT is around 86% with a similar torque like MT. The HST (Hydrostatic Transmission) has a low efficiency of less than 70% and comparatively costly [2]. In the case of HMT (Hydro Mechanical Transmission), the overall efficiency is approximately 80~85% [2,7], and smoothly transmits the power, which applies to the high-power vehicles. Therefore, a PST model was proposed in this study.

The PST is a precision technology of a tractor because the PST is operated by the hydraulic proportional valve that is frequently used in the autonomous industries to control or operate precisely. The proportional valve also uses an electric control system to engage the clutch to enhance power delivery, driving comfort, and being economically efficient [8]. The PST has equipped with a wide range of gear stages without a loss (or minimum) of power during delivery from engine to driving axles [1]. That is why the PST can apply from low power to high power vehicles and also getting popularity as a precise technology.

The PST has two or more wet clutches, which are connected with a hydraulic circuit regulating the pressure by the hydraulic proportional valve [9,10,11]. For decades, the researchers are mainly concerned to minimize the energy loss during clutching, and smooth and fast clutching considering driving comfort [12]. In general, proportional valve is used as flow actuators for hydraulic pistons in a higher level of control loops and for controlling the larger hydraulic valves directly [13,14]. Many efforts have been initiated to implement proportional valves in the agricultural machinery for performance improvement. Several companies, such as John Deere, Claas, Kubota, and Yanmar have applied proportional valves to their agricultural machines for precise performance. Raikwar [15] adapted proportional valve to the tractor power shuttle system due to control the pressure for clutch actuation. It was reported that the proportional valve was performed to control the engage and disengage of the clutch. As the PST is performed by the hydraulic pressure, there are several factors to design the PST model. The scholars’ attempt to find out the design factors for a clutch engagement precisely considering engagement time, smoothness of the gear shifting, friction, low operating force, and easy and flexible engagement of the clutch. The most important factors are the sliding velocity [16], inertia [17], and heat generation [18].

The sliding velocity is the relative speed between the friction plates that occurs during the engagement of the clutch. Generally, the sliding velocity is proportional to the contact surface, as a result squeezing out due to metal-metal contact and consequently scoring [19]. According to He [20], the rolling and sliding are in a relationship with each other at the time of the clutch engagement. Dearn [21] reported that the contact point (pitch point) of clutch engagement is changed for the sliding velocity, which is caused between two metal surfaces for applying loads, stresses, and friction forces. The position of pitch point differs from the clutch engagement time. Ompusunggu [22] reported that transmission fluid in the clutch can reduce the sliding friction coefficients. Therefore, the sliding velocity is considered an important factor to design the transmission to avoid a serious accident.

Moment of inertia is the resistant load of an object to change its rotational motion. Generally, the moment of inertias of both the driving and driven end is converted into the transmission output. In this process, the engine output, input shaft of the transmission, intermediate shaft, and output shaft of transmission are connected to inertia [23]. The clutch engagement process allows a high inertia load to start with small power [24]. Gao [25] reported that the clutch engagement becomes faster with a lower moment of inertia than with a higher moment of inertia. Holgerson [12] also stated a similar assumption for clutch engagement. It means the sliding distance for transmission should be smaller for a certain engagement time with a specified moment of inertia. The reviewed literature revealed that the moment of inertia is a crucial parameter to design a clutch for increasing transmission efficiency, which means the shifting of transmission.

Another important factor is the heat generation between the friction plates during the clutch engagement. The heat generated by friction causes clutch failure for a lengthy time [26,27]. The generated heat can change the friction behavior among the clutch plates by conversing the heat into the thermal stresses [28]. The changed friction behavior has a greater tendency to judder and causes uneven contact with friction plates [29,30]. Also, the generated heat during the clutch engagement is the main reason for the sliding velocity and coefficient of friction that changes the interfacial slip and pressure and leads to slow engagement [31,32]. Therefore, to design a reliable transmission system and improve the optimal performance, it is essential to have a clear understanding of the effect of the heat generation by friction during the clutch engagement in the clutch system.

The above literatures revealed that the sliding velocity, moment of inertia, and the clutch engagement pressure are the important factors for a hydraulic clutch pack. Ompusunggu [22] stated that the clutch engagement is directly affected by the friction coefficients of the friction plates, sliding velocity of the clutch plates, the applied pressures to engage the clutch, and the temperatures of the transmission oil. Lin [33] reported that during engagement process of mechanical clutch, the high thermal heat is generated due to friction between plates within a short time, which caused the sliding. Cui [34] stated that investigation of thermal behavior is comparatively complex because it causes thermos-elastic-plastic deformation by friction. In case of the hydraulic clutch pack, the hydraulic pressure which is applied to perform the clutch pack, generated by the hydraulic oil flow [35]. The flow rate of the hydraulic oils depends on the viscosity of the oils. According to the thermodynamics zeroth law [36], if two friction plates are both in thermal equilibrium with the transmission oil nearby then they are in thermal equilibrium with each other. It means the heat generated by friction is transmitted to the transmission oil and increased temperature of the oils. That is why the thermal heat generated between friction plates is considered equivalent to the temperature of the transmission oil. On the other hand, the hydraulic proportional valve is highly nonlinear due to temperature [37]. The temperature of the transmission oil is highly related to the change of oil viscosity [38]. Javalagi and Singireddy [39] stated that the performance of the hydraulic system varies based on the transmission oil viscosity. However, it is easy to investigate the oil temperatures using low-cost temperature sensors available in the market. Therefore, the temperature of the hydraulic oils is considered an important variable in this study.

Many scholars have been conducted researches on the sliding velocity, the moment of inertia, engagement pressure for mechanical clutch based on the thermal temperature of the friction plates. However, very few researchers were considered the effect of transmission oil temperatures on the engagement pressure, especially for the hydraulic clutch. Therefore, the design factors and variables should be studied to identify the most affected design factors of the hydraulic clutch. The nobility of this study is the temperatures of the transmission oil were used instead of the generated heat by friction to determine the most affected factors and variables of hydraulic clutch pack. The main reason is the viscosity of the transmission oil depends on the temperature of the oil, which affects the supplied pressure of the proportional valve [40,41] that is used as the engagement pressure in the clutch. As the clutch engagement is performed by the hydraulic pressure, the performance of the PST depends on the viscosity of the transmission hydraulic oil [39,42]. Therefore, the design factors and variables for the hydraulic clutch pack are essential to determine based on the temperature of the transmission oil.

This study was focused to develop a hydraulic clutch pack simulation model of a PST for the agricultural tractor to improve the clutch transmission by the determination of the factors that affect the power transmission directly. Therefore, the specific objectives of this study are as follows: (i) to develop a simulation model of a hydraulic clutch pack for the PST; (ii) to analyze the factors that affect the hydraulic clutch pack engagement time; and (iii) to analyze the clutch engagement pressures at different temperatures of the hydraulic oils.

## 2. Materials and Methods

### 2.1. Specifications of PST Tractor

The tractor used in this study was manufactured by Daedong company, Korea. The dimension of the tractor (Length × Width × Height) were 3810 × 1960 × 2730 mm. The rated power of the engine was 95 kW at the rated rotational speed of 2000 rpm. The transmission was full power-shift with a combination of 16 × 16 gear stages for both forward and reverse directions. In this study, the sliding friction coefficient was selected 0.09 to determine the sliding velocity of the clutch pack [33]. The maximum hydraulic pressure of the proportional valve is 30 bar to engage the clutch [4]. The specifications of the PST tractor are provided by the manufacturer company (Daedong, Daegu, Korea), and also collected from previous literatures conducted on power-shift transmission. The specifications of the PST tractor were listed in Table 1.

### 2.2. Powertrain and Configurations of the Clutch Pack for the PST

**Figure 1:**
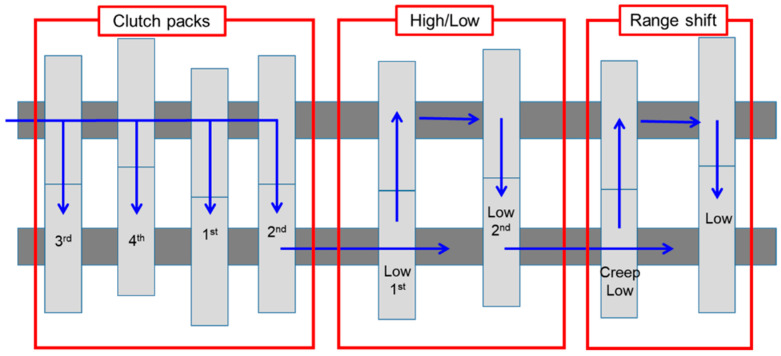
The schematic diagram of the gear stages of PST.

**Figure 2:**
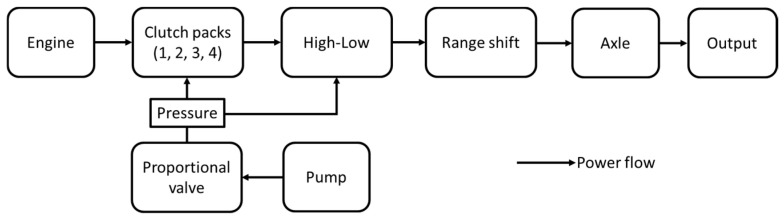
The block diagram of the PST configuration.

The powertrain of the PST model used in this study was composed of 4 clutch packs, high/low, and range shift. The schematic diagram of the powertrain of the PST is shown in Figure 1. In this study, the PST has been configured with 16 × 16 for both the forward and reverse gear stages. The engine power is delivered through clutch packs dealing with high/low by decreasing or increasing speed, and range shift to the driving axle. The range shift helps to perform various agricultural works based on load levels. The block diagram of the PST configuration is shown in Figure 2. It is observed that the clutch packs are the key component of the transmission. The clutch packs make the connection between the shaft from the engine and the shaft which turns the driving wheels. The clutch packs allow the vehicles to change speed and stop completely without turning off the engine. To stop the vehicle, the connection between the engine and the driving wheels need to break temporarily that is performed by the clutch packs. Each clutch pack of the PST is performed by the hydraulic pressure, which is supplied from the individual proportional valve. Therefore, the design of the clutch pack for the PST of the agricultural tractor is the main concern in this study.

### 2.3. Factors and Variables of the Hydraulic Clutch Pack

**Figure 3:**
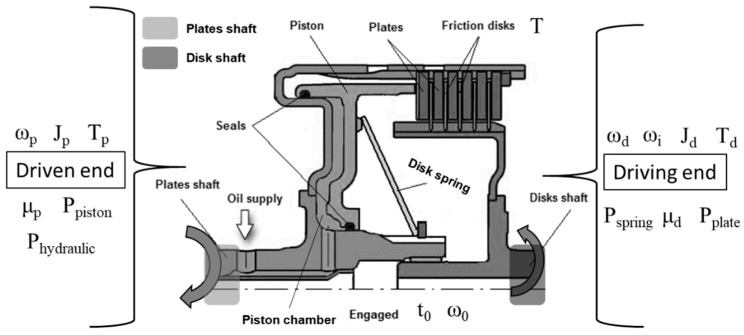
The cross-sectional view of the clutch pack for the PST.

There are several factors and variables to design the hydraulic clutch pack operated by the hydraulic pressure for the PST tractor. Among them, the most important factors are sliding velocity and moment of inertia, which are highly influenced by the heat generated in the friction plates during clutch engagement. It also causes a higher engagement time. Therefore, the sliding velocity, moment of inertia, and clutch engagement pressure are essential to design optimally, especially for the hydraulic pressure operated clutch pack. The cross-sectional view of a hydraulic clutch pack for the PST of an agricultural tractor is shown in Figure 3. In this figure, the hydraulic pressure supplied from port A of the proportional valve acts into the piston chamber and the piston rod pushes the clutch plates to engage the clutch. That is why this pressure is called the engagement pressure of the clutch.

#### 2.3.1. The Sliding Velocity of the Clutch

The clutch engagement pressure is applied to the friction plate when the clutch is being engaged. The procedure of dynamic clutch engagement should be studied to estimate the clutch relative sliding velocity of the PST, which is shown in Figure 3. After that, the angular velocity of both driving and driven end should be linearly assumed. The mathematical formula to calculate the angular velocities of both driving and driven end [43] are as following Equations (1)–(3). Equation (3) is the relative sliding velocity between friction plates at the time of clutch engagement.

(1) $$\omega_{d}=\omega_{i}-\frac{\omega_{i}-\omega_{0}}{t_{0}}\times t,$$

(2) $$\omega_{p}=\frac{\omega_{0}}{t_{0}}\times t,$$

(3) $$\omega =\omega_{d}-\omega_{i}=\omega_{i}\;(1-\frac{t}{t_{0}}),$$

where ω_d_, and ω_p_ are the angular velocity of driving and driven end (rad/s), respectively; ω_i_ is the initial angular velocity of driving end (rad/s); ω_0_ is the angular velocity of both driving and driven end at *t*_0_ (rad/s); $t_{0}$ is the engagement time of clutch (s), and *t* is the time (s) (0 < *t* ≤ $t_{0}$).

#### 2.3.2. Moment of Inertia

The moment of inertia of both driving and driven end of the clutch pack should be considered to analyze the torque of both ends. The moment of inertia of the clutch pack (Figure 3) is the equivalent moment of inertia of both driving and driven end of the clutch pack [33]. The differential equation of moment of inertia at the driving end during clutch engagement is as following Equation (4).

(4) $$T_{d}-T=J_{d}\;(\frac{d\omega_{d}}{dt}),$$

where T_d_ is the driving torque (Nm); T is the friction torque between friction disks during the engagement (Nm), and J_d_ is the equivalent moment of inertia at the driving end (kg·m^2^).

The differential equation of the moment of inertia at the driven end during engagement of the clutch is as below Equation (5).

(5) $$T-T_{p}=J_{p}\;(\frac{d\omega_{p}}{dt}),$$

where T_p_ is the resisting torque (Nm), and J_p_ is the equivalent moment of inertia at the driven end ($\mathrm{kg}\cdot m^{2}$).

It is assumed that the applied engagement pressure (P_plate_) is uniformly distributed between the plate and friction disks. Therefore, the friction torque (T) is expressed as Equation (6).

(6) $$T=N\times \frac{2\pi µ}{3}\times (R_{o}^{3}-R_{i}^{3})P_{\mathrm{plate}},$$

where N is the number of the engaged surfaces; µ is the coefficient of friction; P_plate_ is the retraction pressure of the clutch plate (N/m^2^), and *R*_i_ and *R*_o_ are the inner and outer diameters of the friction disks (m), respectively.

The driving end of the clutch pack is operated by the engine and driving torque, which can be calculated using the following Equation (7).

(7) $$T_{d}=T_{\max}-\frac{T_{\max}-T_{p}}{{\left(n_{p}-n_{\max}\right)}^{2}}(n_{\max}-n_{e}{)}^{2},$$

where *T*_max_ is the engine maximum torque (Nm); *T*_p_ is the corresponding maximum torque (Nm) at the maximum power; *n*_max_ is the engine maximum speed (rpm); *n*_p_ is the corresponding maximum speed (rpm) at the maximum power, and *n*_e_ is the engine speed (rpm) [*n*_e_ = (60/2π)ω_e_].

#### 2.3.3. Clutch Engagement Pressure

The hydraulic clutch pack assembly of the PST is presented in Figure 3. The engagement of the clutch is performed by regulating the proportional valve pressure that is called the compressor pressure. This compressor force is occurred by the piston through the clutch plate [44,45], which is expressed by the following Equations (8) and (9).

P_piston_ = µ_p_ × P_hydraulic_, (8)

P_plate_ = µ_d_ × P_hydraulic_, (9)

where P_piston_ is the compressor pressure of the clutch piston (bar); P_plate_ is the compressor pressure of the clutch plate (bar); P_hydraulic_ is the hydraulic pressure to engage the clutch (bar), and µ_p_ and µ_d_ are the dynamic friction coefficient of the clutch piston and plate, respectively.

The clutch is disengaged by the retraction force of spring, which can be expressed as Equations (10) and (11).

P_piston_ = µ_p_ × P_spring_, (10)

P_plate_ = µ_d_ × P_spring_, (11)

where P_piston_ is the retraction pressure of the clutch piston (bar); P_plate_ is the retraction pressure of the clutch plate (bar); P_spring_ is the pressure to disengage the clutch (bar) and µ_p_ and µ_d_ are the dynamic friction coefficient of the clutch piston and plate, respectively.

### 2.4. Simulation Model of the Hydraulic Clutch Pack for the PST

**Figure 4:**
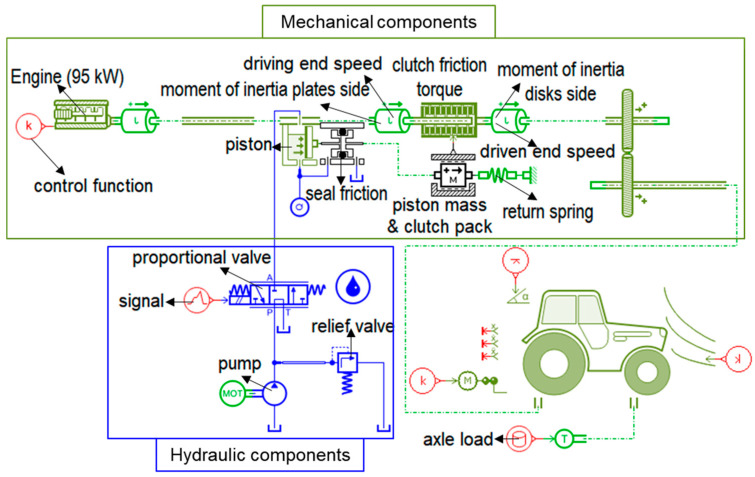
The simulation model of the hydraulic clutch pack for the PST.

The PST which is composed of main driving shift (4 clutch packs), 2 high-low clutch, and 2 stages of range shifts in the powertrain, is the entire PST model (Figure 2). This study is focused on the determination of design factors and variables for the PST clutch packs. Also, the clutch packs and high-low clutches which consist of several mechanical components, are the same structure. Therefore, the simulation model was simplified to determine the design factors and variables for the PST clutch pack, which is shown in Figure 4. The clutch pack is operated by the hydraulic pressure and the hydraulic pressure regulates the clutch engagement by the proportional valve. To be characterized by real machinery, the axle load and tractor specifications were applied in the tractor model. The axle load was estimated using the traction prediction model [46,47].

In this study, the simulation model of the clutch pack for the PST has developed by the commercial simulation software namely LMS AMESim (version 16, SIEMENS AG, Munich, Germany), which is operated by the 95 kW engine. In this model, “k” is the control function, which is range of 0 to 1. The “k” value 1 means the engine can operate 100% of throttle. Therefore, throttle level can easily be controlled using “k” value. Moment of inertia for both plate and disk sides was determined based on the friction torque of the clutch.

The sliding velocity (driving and driven) was measured at inertia of both sides of the clutch. The seal friction is a coefficient that is either the coefficient of static friction or the dynamic friction. The seal friction coefficient makes a relationship between the friction force of two objects that resists an object to move smoothly. The return spring is performed to disengage the clutch. The step signal was used for valve opening. The relief valve pressure was set at 50 bar to conduct the simulation.

### 2.5. Simulation Procedure

#### 2.5.1. Engine Map and Travel Speed

The 95 kW engine was used in this study. The engine static torque is a function of engine speed and throttle opening [10]. The mathematical model of engine torque is in Equation (12).

(12) $$T_{e}=\int \left(n_{e},\alpha \right),$$

where T_e_ is the engine torque (Nm); *n*_e_ is the engine speed (rpm), and α is the engine throttle opening (%).

**Figure 5:**
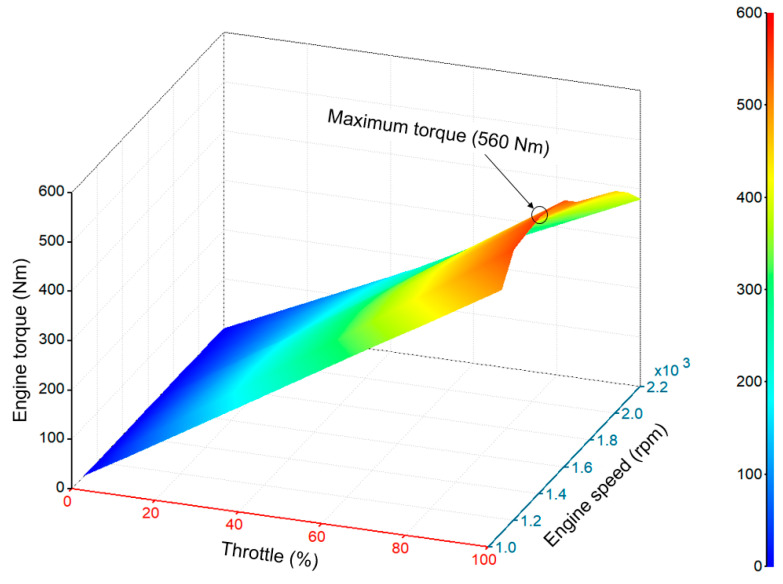
The 95 kW engine characteristics map used in this study.

The engine torque was calculated at each throttle (%) using Equation (12), which was applied to develop the static engine map used in this study is shown in Figure 5, whereas the maximum torque was 560 Nm at 1400 rpm for 33.33% of throttle opening. The simulation was conducted using the developed engine map.

#### 2.5.2. Input Conditions

**Figure 6:**
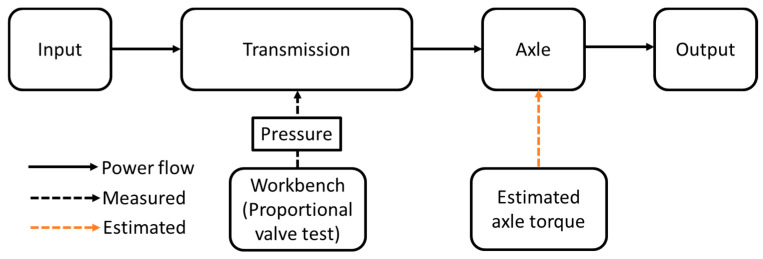
The schematic diagram for applied simulation conditions in this study.

The schematic diagram of the simulation procedure was shown in Figure 6. The supplied pressure of the proportional valve, which is considered as equal to the engagement pressure because the supplied pressure at port A of the proportional valve acts on the piston to engage the clutch. Therefore, a test bench was constructed to measure the supplied pressure at port A of the proportional valve and applied to the simulation model. The proportional valve pressure was measured based on the different temperatures of the hydraulic oils. The estimated axle load was also applied to the tractor model. The simulation parameters such as the sliding friction coefficients were calibrated using the measured proportional valve pressures. The clutch pack design condition was set 1.20 s as the maximum engagement time for PST in this study [38].

#### 2.5.3. Entire Methodology

**Figure 7:**
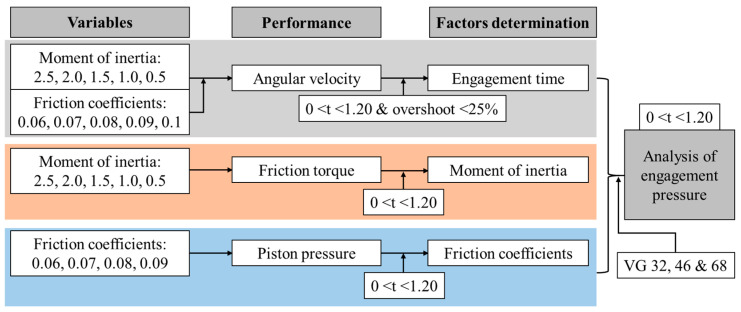
The overall procedure of this study.

The overall simulation process of this study was shown in Figure 7. First, the shifting time was determined for various levels of moment of inertia and friction coefficients on the basis of angular velocity. The designed engagement time was selected 0 < t ≤ 1.20 s. Second, the moment of inertia was determined by performing the clutch friction torque. Third, the piston pressures were performed based on various levels of the friction coefficients and the clutch engagement time. Finally, the engagement pressures of the clutch were performed to verify the selected engagement time, moment of inertia, and friction coefficients with respect to the measured proportional valve pressure, estimated axle torque, and real tractor specifications. The clutch engagement pressures were analyzed at various temperature levels for three hydraulic oils (VG 32, 46, and 68).

### 2.6. Proportional Valve Test Bench and Experimental Procedure

**Figure 8:**
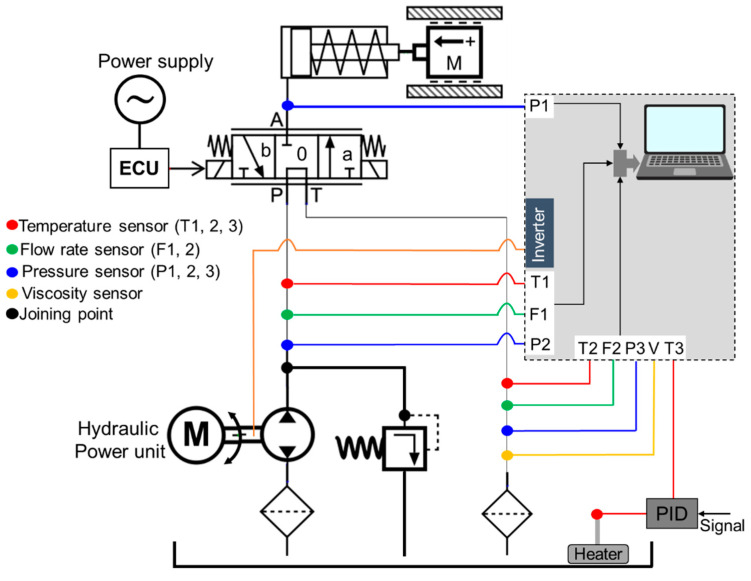
The scheme of the proportional valve test.

The scheme of the proportional valve test was shown in Figure 8. The sensing data was controlled by control unit (CU) and the electronic control unit (ECU) supplied the controlled current to the proportional valve. The pressure sensor (PSHH0025BAIG, Sensys, Ansan-Si, Korea) was installed to measure the pressure of the proportional valve (PDMC05S30A-50-C-N-20, HYDAC, Sulzbach, Germany) at three points (inlet, outlet, and the inside of each proportional valve). The supplied pressure of the proportional valve at port A was measured by test bench is applied as the engagement pressure of the piston supplied from the proportional valve in this simulation model. There were also installed three temperatures sensors at inlet and outlet of the proportional valve, and hydraulic tank. A controlled temperature sensor was installed in the hydraulic tank to measure continuous temperature changes of the hydraulic oils. Flow rate (VZ040GGV32I00S, Sika, Stuttgart, Germany) and viscosity (YTS31, Yateks, Shenzhen, China) sensors were installed to monitor the flow and viscosity changes of the oils at different temperatures. However, the temperatures and pressures sensors data were used in this study. The speed of the hydraulic pump was controlled using inverter (SV-iG5A, LS Industrial System, Anyang, Korea). The data acquisition (DAQ) was developed by Python (ver.:3.9.0). NI (USB 6009) DAQ was used to measure the sensing data. The specifications were listed in Table 2.

**Figure 9:**
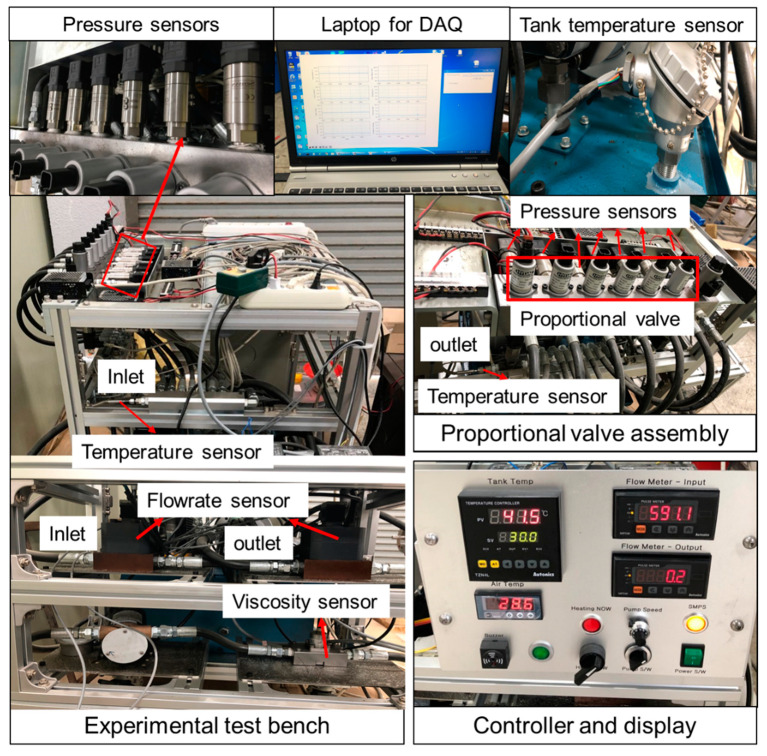
The experimental bench for the proportional valve test.

The engagement pressures are influenced by the heat generated in the friction plates [33]. For being a limited test condition of the clutch temperature generated by the friction, the hydraulic oil temperatures were considered to measure the supplied pressure of the proportional valve at the pump rotational speed of 2000 rpm. The temperatures were determined 25, 40, 60, 80, and 100 °C for measuring the supplied pressure of the proportional valve because the highest temperature of the hydraulic oils of the agricultural machinery is 100 °C [48]. The heating system was also installed in the hydraulic tank to increase the oil temperature operating by heater switch. The experimental bench for the proportional valve test was shown in Figure 9.

The various types of hydraulic oils were used because the viscosity of the hydraulic oil is inversely proportional to the temperature and the low pressures occur at low temperatures due to high viscosity [49]. Actually, the flow rate of the hydraulic oils highly depends on viscosity. When the viscosity of the oils decreases the flow rate increases. As a result, the pressure also increases meaning is that the temperature, flow rate, and pressure are inversely proportional to the viscosity of the hydraulic oils [40]. The experiment was conducted by three ISO standard hydraulic oils namely Kixx RD Z (VG 32, 46, 68). The designed factors were determined using the hydraulic oils VG 32, 46, and 68 in this study. The specifications of the hydraulic oils are listed in Table 3.

### 2.7. Statistical Analysis

The clutch engagement pressures of a hydraulic clutch pack for the PST that is supplied from proportional valve port A acts on piston rod to push the clutch plates. The engagement pressures were analyzed based on various temperature levels of the transmission oils (VG 32, VG 46, and VG 68) because temperatures directly affect the engagement pressures. In this study, the designed factors were determined for the hydraulic oils of VG 68, and VG 32, VG 46, and VG 68 were applied to verify the designed factors. The statistical analysis, one-way ANOVA, and Duncan’s multiple range test (DMRT) were performed to analyze the significance of the clutch engagement pressure for each temperature level of the hydraulic oils. The software used for the analysis was IBM SPSS Statistics (SPSS 25, SPSS Inc., New York, NY, USA).

## 3. Results and Discussion

### 3.1. Proportional Valve Pressure

**Figure 10:**
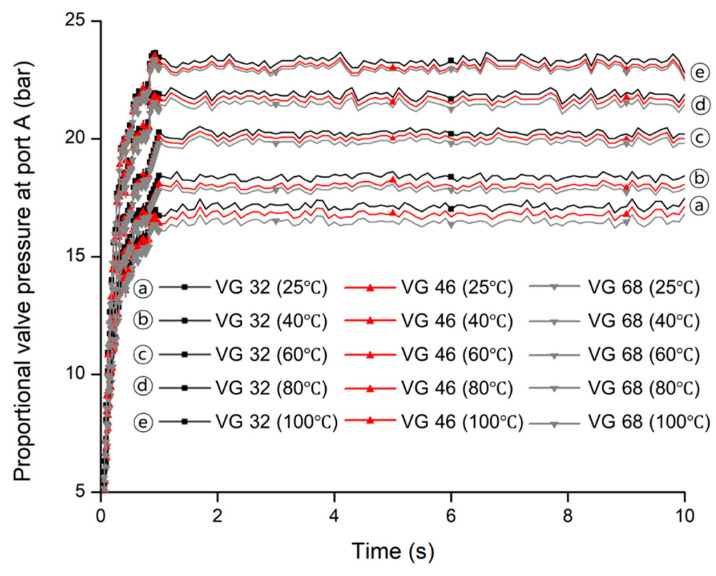
The pressures of the proportional valve at port A for the hydraulic oils measured using test bench at: (**a**) 25 °C; (**b**) 40 °C; (**c**) 60 °C; (**d**) 80 °C; and (**e**) 100 °C.

The proportional valve pressures at port A were measured for three hydraulic oils (VG 32, VG 46, and VG 68), which is shown in Figure 10. (a), (b), (c), (d), and (e) are five temperature levels (25, 40, 60, 80, and 100 °C) for three hydraulic oils (VG 32, VG 46, and VG 68). At each temperature level, the pressure was similar trend and maximum pressure was almost the same for all hydraulic oils. It was observed that the maximum pressure has big differences among the temperature levels. It was noticed that the proportional valve pressure increased gradually and became steady within 1.2 s for all hydraulic oils.

The highest and lowest maximum pressures were found at temperature levels (e) and (a) at 100 °C and 25 °C, respectively for all hydraulic oils. As that the maximum pressures of the proportional valve were variable for different hydraulic oils at different temperatures, meaning that the supplied pressure of the proportional valve varied according to the viscosity of the hydraulic oils because the viscosity of the hydraulic oils is inversely proportional to the temperatures of the hydraulic oils. Those measured data were applied to the simulation model to determine and optimize the design factors of the clutch pack for the PST. Actually, this pressure acts into the piston chamber. As a result, the piston rod pushes the clutch plates to engage the clutch. That is why the supplied pressure at port A of the proportional valve is called the engagement pressure in this study.

### 3.2. Analysis of the Factors

#### 3.2.1. Sliding Velocity

**Figure 11:**
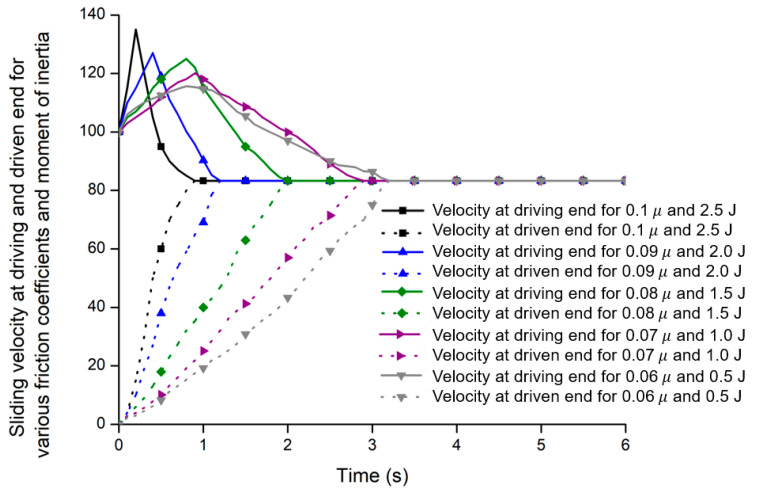
The sliding velocity of the clutch at driving and driven end.

The sliding velocity is highly related to the heat generated in the friction plates during the clutch engagement, which is equivalent to the hydraulic oil temperatures based on the thermodynamics zeroth law [36]. The sliding causes the time delay to engage or disengage the clutch that can occur in a serious accident. Figure 11 shows the sliding velocity of the clutch packs for both driving and driven end during the engagement, which caused the speed difference between engine and drivetrain by the heat generation in the friction plates. In general, the sliding velocity at driving end (inertia before clutch) will gradually decrease and the sliding velocity at driven end (inertia after clutch) will gradually increase. Finally, both velocities will be stable within designed engagement time (1.20 s) near the reference sliding velocity (80 rad/s).

It was noticed that the maximum peak velocities were found for 0.1 friction coefficient (μ) and 2.5 moment of inertia (J) for diving end. It was observed that the clutch engagement time is inversely proportional to the overshoot. The percent overshoot is the function of damping ratio, which is a nonlinear character of the proportional valve. Ding [50] stated that the damping ratio is a critical factor to make the hydraulic system unstable. The damping ratio is one of the main reasons for the overshoot [40]. The engagement time was 1.20 s at friction coefficient (0.09) and moment of inertia (2.0), which was the design criteria [38]. Also, the faster engagement time might be found at lower sliding friction coefficients but the shifting shock of the clutch pack is inversely proportional to the shifting time [51]. In case of friction coefficient (0.1) and moment of inertia (2.5), the maximum overshoot was found 25.93%, which exceeds the critical criteria (25%) [40]. The performance of the sliding velocity of the PST was shown in Table 4. Therefore, the engagement time was determined 1.20 s based on the sliding friction coefficient of 0.09.

#### 3.2.2. Moment of Inertia

**Figure 12:**
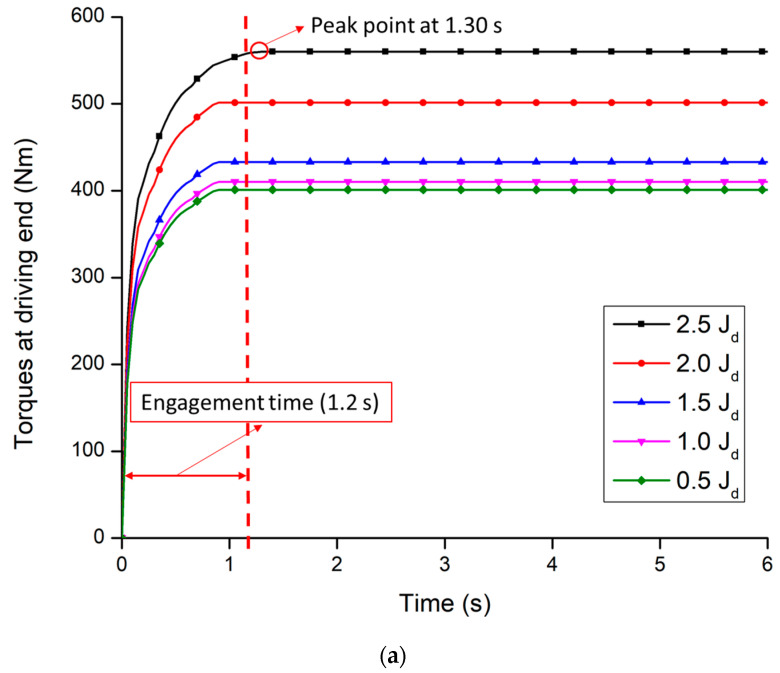

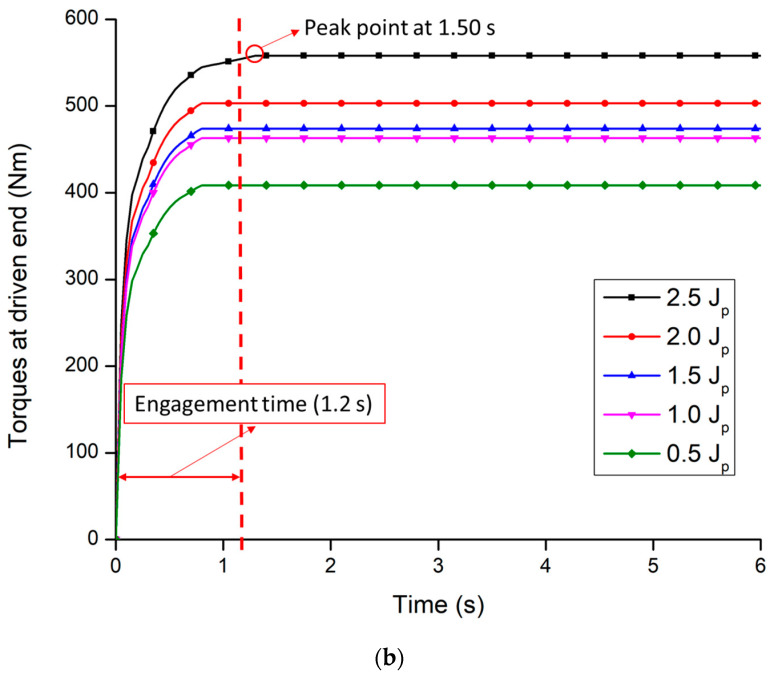
The torque of the clutch at a different moment of inertia: (**a**) Driving end; (**b**) Driven end.

The torque of both driving and driven end influence by the moment of inertia because the moment of inertia is highly affected by the heat generated in the friction plate during clutch engagement. Figure 12a,b shows the torques of the clutch at a different moment of inertia for both driving and driven end. It was observed that the torques of the clutch for both ends were directly proportional to the moment of inertia. However, the maximum torque was also found between the designed engagement time at the moment of inertia (J_d_) less than or equal 2 kg·m^2^, which accounts for a range of 0.95 to 1.02 s for driving end. It was noticed that the time of maximum torque was slightly higher than the design conditions 1.2 s when the moment of inertia was over 2 kg·m^2^. The time to be stable the driving end torque was 1.30 s at the moment of inertia of 2.5 kg·m^2^, which is shown in Figure 12a. In the case of the driven end (Figure 12b), the time of maximum torque, which was found over the design value, was 1.50 s when the moment of inertia was over 2 kg·m^2^. However, the times of maximum torques were below the 1.20 s (range: 0.93 to 0.98 s), when the moment of inertia was less than or equal 2 kg·m^2^.

The time required for maximum torque is listed in Table 5. The results indicate that the moment of inertia cannot be applicable over 2 kg·m^2^ because it takes over the designed clutch engagement time. On the other hand, when the moment of inertia is lower than 2 kg·m^2^, the clutch engagement time was comparatively lower. The transmitted torques from the engine to the driven end were also lower. The transmission purposes to deliver the maximum engine torque through the clutch pack. The performance of the clutch depends on the transmission of the engine torque to the driven end [52,53]. Therefore, the optimal moment of inertia was 2 kg·m^2^ because the maximum transmitted torque was 502.90 Nm at the driven end within the designed engagement time, whereas the engine’s maximum torque was 560 Nm.

#### 3.2.3. Clutch Engagement Pressure

The clutch engagement pressures is directly proportional to friction coefficients ($\mu_{p}$). The range of the friction coefficient was selected from 0.06 to 0.09 according to the friction plates used in this study. Yan [54] also applied the friction coefficient range of 0.05 to 0.09 for the simulation model of synchronizer’s friction pair for transmission.

**Figure 13:**
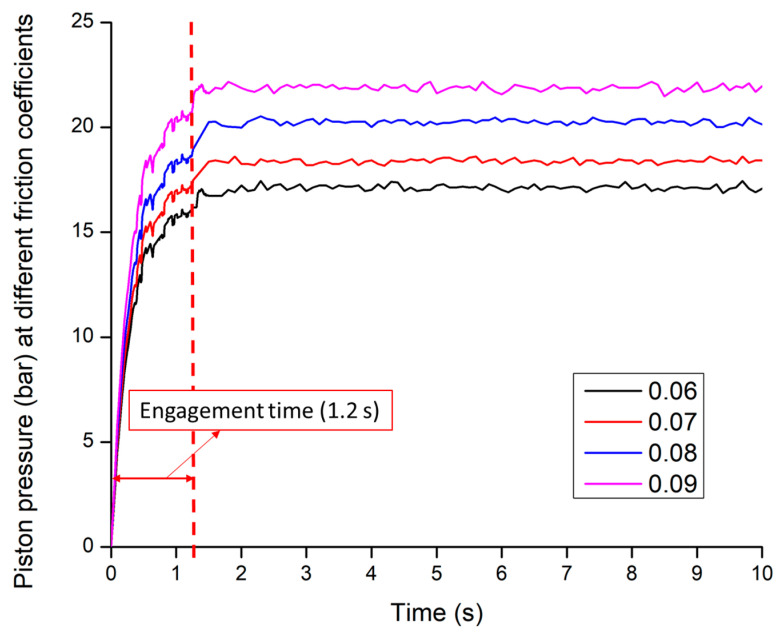
The piston pressures at different friction coefficients.

It was observed that the piston pressure at the friction coefficient of 0.09 was only satisfied with the designed engagement time (1.20 s). The engagement time was found 1.20 s at 0.09 of friction coefficient. In other cases, the piston pressures took comparatively higher time to engage the clutch. The piston pressures were shown in Figure 13. It was also observed that the engagement time is inversely proportional to the friction coefficients. The piston pressure took the maximum time at the friction coefficient of 0.06, which was 1.55 s. The piston pressures at different friction coefficients of the clutch with the engagement time were listed in Table 6. The statistical analysis shows that the clutch engagement pressures have a highly significant difference among the friction coefficients.

### 3.3. Analysis of the Engagement Pressure

The engagement pressures are influenced by the heat generated in the friction plates during the engagement. The hydraulic oils (VG 32, VG 46, and VG 68) at the different temperatures were selected to verify the designed factors. To analyze the engagement pressure, the selected sliding friction coefficient (0.09), moment of inertia (2 kg·m^2^), and friction coefficient of both driving and driven end (0.09) were set, and conducted the engagement pressure of VG 32, 46, and 68 at different temperatures of 25, 40, 60, 80, and 100 °C.

**Figure 14:**
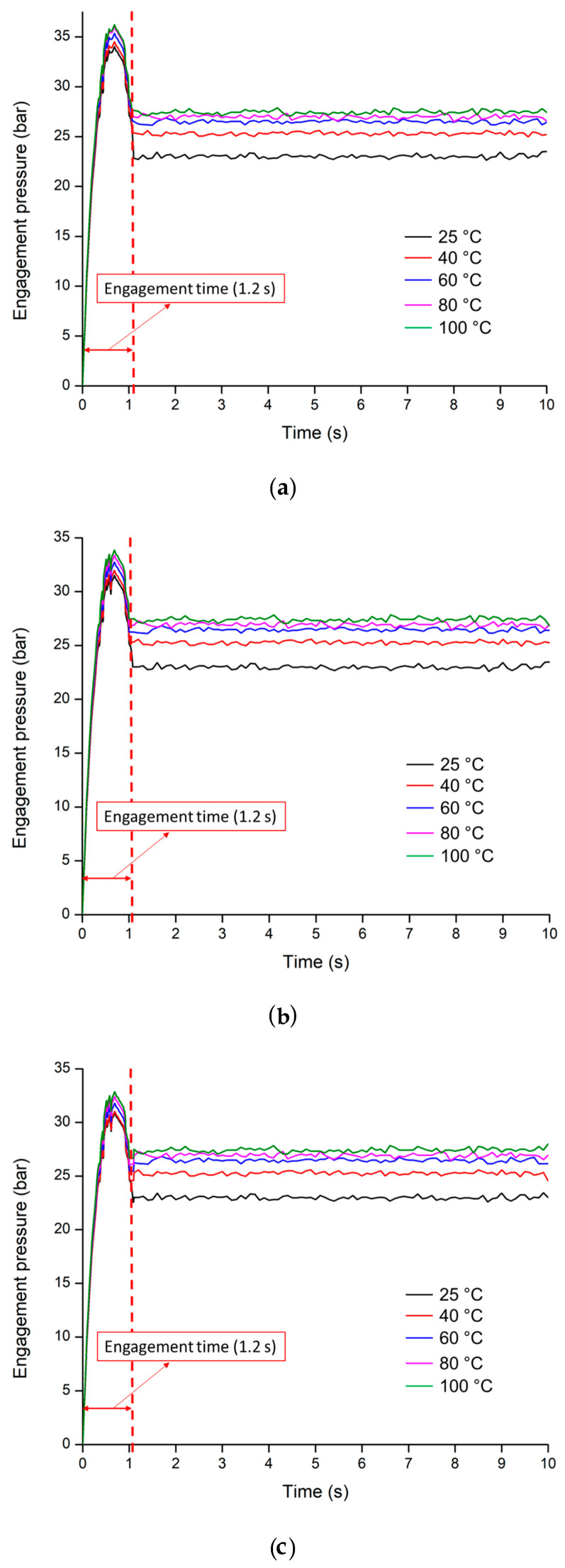
The engagement pressures of the clutch at different temperatures of the hydraulic oils: (**a**) VG 32; (**b**) VG 46; (**c**) VG 68.

The results show that the clutch engagement pressures at the piston chamber were almost steady with the increasing of time, especially after designed conditions (1.20 s), which is shown in Figure 14a–c. It was also noticed that the overshoot of the clutch engagement pressures is proportional to the temperatures because the shifting time is inversely proportional to the shifting shock of the clutch pack [35]. It was found that the clutch engagement times were constant at all temperatures (25, 40, 60, 80, and 100 °C) for all the hydraulic oils, accounting for 1.20 s. The maximum peak point of the pressure was found at 100 °C, accounts for approximately 36.22 bar for VG 32, which is shown in Figure 14a. The lowest peak point was around 34.01 bar at 25 °C for the hydraulic oil of VG 32. The maximum and minimum peak pressures for VG 46 were calculated around 33.85 and 31.49 bar at 100 and 25 °C, respectively, which is shown in Figure 14b. In case of VG 68, the maximum and minimum pressure peaks were 32.86 and 30.87 bar for 100 and 25 °C, accordingly.

The clutch engagement time was found constant for each temperature level of the hydraulic oils, accounting for 1.20 s. It was observed that the clutch engagement pressures were variable at different temperatures for all hydraulic oils. The statistical analysis proved that the engagement pressures have a highly significant difference among the temperature levels of the hydraulic oils. In case of low temperatures (25 and 40 °C) for all hydraulic oils, there was no significant difference. It was also noticed that the engagement pressures have no significant difference among different hydraulic oils at 25 °C. The reason was the higher viscosity of the hydraulic oils. At 100 °C for VG 46 and 68, the statistical results show that there was no significant difference among them because of the low viscosity of the hydraulic oils. The statistical analysis results were listed in Table 7.

These results indicate that the design of the sliding velocity means the sliding friction coefficients, moment of inertia, and friction coefficient for both driving and driven end were optimized that can maintain the clutch engagement time. It means that the measured pressures were synchronized by adjusting the sliding velocity, moment of inertia, and friction coefficient with the clutch engagement pressure that can ensure the clutch engagement on time within the designed time. However, the engagement pressures have a significant difference at different levels of temperatures of the hydraulic oils after 1.20 s because the determined factors cannot compensate for the nonlinearity effect of the hydraulic system. Li [55] stated that the design factors can satisfy the engagement time but the nonlinear characteristics of the hydraulic system cannot compensate by adjusting the sliding velocity, friction coefficient, and moment of inertia. Therefore, a control algorithm is needed to control the clutch engagement pressure.

## 4. Conclusions

This study was focused on the determination of the most affected design factors and variables of the clutch pack for the power-shift transmission (PST) of an agricultural tractor. The simulation model of the clutch pack for PST was developed using the powertrain and configurations of the real PST tractor. The sliding velocity, a moment of inertia, and clutch engagement pressure were mathematically designed for the clutch pack based on the transmission oil temperatures. The ranges of temperatures were 25 to 100 °C. The supplied pressure of the proportional valve was measured using a proportional valve test bench, which was used as input parameters. The friction coefficients were calibrated by the measured pressure data. Finally, the engagement pressures were analyzed to ensure maximum power transmission. The overall findings of this study are listed below:

1. The results were shown that the sliding velocity at the driving end decreased from 100 rad/s at 0.8 s, whereas the sliding velocity at the driven end rose from zero at 0.8 s and the engagement procedure took 1.20 s, whereas the angular velocity was 83.28 rad/s for the sliding friction coefficient of 0.09.
2. It was noticed that the peak torque increased with the increase of the moment of inertia for both driving and driven end. The moment of inertia could not be applicable over 2.0 kg·m^2^ because it took over the designed engagement time. In other cases, it was noticed that the peak torque was comparatively lower at 1.5 kg·m^2^ moments of inertia. Therefore, the moment of inertia was selected as 2 kg·m^2^ for both driving and driven end considering the delivered peak torque.
3. It was observed that the clutch engagement time is inversely proportional to the friction coefficient of driving and driven end. The engagement time was satisfied with the designed engagement time for the friction coefficient of 0.09, accounting for 1.20 s. In other cases, the piston pressures required comparatively higher time to engage the clutch. The highest time was found around 1.55 s for the lowest friction coefficient of 0.06. Therefore, the friction coefficient of both the driving and driven end was selected 0.09.
4. It was also observed that the engagement pressures were almost steady with the increasing of time, especially after 1.20 s. It was found that the clutch engagement times were 1.20 s at all temperatures of the transmission oils. The maximum peak point of the pressure was found at 100 °C accounts for approximately 36.22 bar. The lowest peak point was around 30.87 bar at 25 °C for the hydraulic oil of VG 68.

In summary, it was observed that the clutch engagement times have no difference at different temperatures for both hydraulic oils. However, the engagement pressures have a significant difference at different levels of temperatures of the hydraulic oils after the 1.20 s. Interestingly, the results indicate that the design of the sliding velocity means sliding friction coefficients, moment of inertia, and friction coefficient for both driving and driven end were optimized that can maintain the clutch engagement time. It was also noticed that the determined factors cannot compensate for the nonlinearity effect of the hydraulic system. Therefore, a control algorithm is needed to control the clutch engagement pressure.

In this study, the sensor fusion method (temperature, pressure, and flow sensor) was used to precisely measure the supply pressure of the proportional valve. This sensor fusion method is believed to be helpful in realizing precision agriculture through minimization of power loss and maximum energy efficiency of agricultural tractors. In the future, the control algorithm will be developed to compensate for the nonlinear effects and to control the engagement pressures, and the shifting shock will be concerned to improve the transmission shifting performance of the hydraulic clutch pack for the PST of an agricultural tractor. The drawback of this study is that it is presented the mathematical and simulation models of the hydraulic clutch pack. It is needed to verify the simulation results by the prototype or real PST tractor performing field operation. However, the important thing is that the determination of the most affected factors was satisfied with the simulation conditions that ensure the clutch engagement on time. Finally, it can be said that this model could apply to the real PST tractor to improve the engagement time based on hydraulic pressures supplied from the proportional valve.

## Figures and Tables

**Table 1:** The specifications of the tractor used in this study.

Items	Parameters	Specifications
Tractor	Length × Width × Height (mm)	3810 × 1960 × 2730
	Weight (kg)	3630
Engine	Rated speed (rpm)	2000
	Rated power (kW)	95
	Rated fuel consumption (kg/h)	22.5
Transmission	Transmission type	Full power-shift
	No. of clutch packs	4
	High-Low	2
	Range shift (Creep)	2
	Gear stages (forward × reverse)	16 × 16
Clutch pack	Clutch	Hydraulic
	Sliding friction coefficient	0.09
	Maximum pressure (bar)	30

**Table 2:** The specifications of the equipment and sensors used in this study.

Equipment/Sensor	Parameters	Specifications
Proportional valve	Company, Country	HYDAC, Germany
	Model	PDMC05S30A-50
	Measuring range (LPM)	20
	Pressure range (bar)	25
Pressure sensors	Company, Country	Sensys, Korea
	Model	PSHH0025BAIG
	Measuring range (bar)	25
	Accuracy	±0.15% FS (RSS)
Flow rate sensors	Company, Country	SIKA, Germany
	Model	VZ040GGV32I00S
	Measuring range (LPM)	0.2 to 40
	Pressure (bar)	315
	Viscosity range (mm^2^/s)	1 to 100,000
	Accuracy	±0.3% of reading
Viscosity sensor	Company, Country	Yateks, China
	Model	YTS31
	Measuring range (mm^2^/s)	500
	Temperature (°C)	−40 to 120
	Accuracy	5% of viscosity
Inverter	Company, Country	LS, Korea
	Model	SV-iG5A
	Frequency (Hz)	60

**Table 3:** The specifications of the hydraulic oils used in this study.

Parameters	Hydraulic Oils		
	VG 32	VG 46	VG 68
Brand (Model)	Kixx (RD Z)		
Density (kg/L) at 15 °C	0.851	0.860	0.865
Kinematic viscosity (mm^2^/s) at 40 °C	31.7	46.2	68.1
Kinematic viscosity (mm^2^/s) at 100 °C	6.36	8.21	10.9
Viscosity index	157	153	151
Pour point (°C)	−45	−42	−42
Flash point (°C)	218	228	234

**Table 4:** The performance of the sliding velocity of the PST.

Parameters	μ	J	μ	J	μ	J	μ	J	μ	J
	0.06	0.5	0.07	1.0	0.08	1.5	0.09	2.0	0.1	2.5
Peak velocity (rad/s)	115.59		120.10		125		127		135	
Engagement time (s)	3.20		2.90		2.00		1.20		0.90	
Overshoot (%)	13.48		16.74		20		21.26		25.93	

**Table 5:** The times of peak torques at a different moment of inertia.

**Parameters**	**Moment of Inertia (J_d_) at the Driving End (kg·m^2^)**				
	**2.5**	**2.0**	**1.5**	**1.0**	**0.5**
Max. torque (Nm)	559.82	501.27	432.91	410.13	401.02
Peak time (s)	1.30	1.02	0.98	0.96	0.95
**Parameters**	**Moment of Inertia (J_p_) at the Driven End (kg·m^2^)**				
	**2.5**	**2.0**	**1.5**	**1.0**	**0.5**
Max. torque (Nm)	557.82	502.90	473.78	462.89	408.43
Peak time (s)	1.50	0.98	0.95	0.95	0.93

**Table 6:** The piston pressures at different friction coefficients.

Parameters	Friction Coefficients $\left(\mu_{p}\right)$			
	0.06	0.07	0.08	0.09
Max. pressure (bar)	17.58	18.89	20.91	22.44
Avg. ± S.D. *	13.85 ^a^ ± 3.99	14.86 ^b^ ± 3.86	16.19 ^c^ ± 4.70	17.88 ^d^ ± 5.13
Engagement time (s)	1.55	1.50	1.42	1.20

^a,b,c,d^ Means within each column with the same lettering are not significantly different at *p* < 0.05 according to Duncan’s multiple range test; * Avg. ± S.D. is the Average ± Standard Deviation.

**Table 7:** The engagement pressures at different temperatures levels of the hydraulic oils.

Hydraulic Oils	Parameters	Temperatures (°C)				
		25	40	60	80	100
VG 32	Max. pressure (bar)	34.01	34.52	35.37	36.05	36.22
	Avg. ± S.D.	25.75 ^a^ ± 7.79	26.53 ^a^ ± 7.81	27.30 ^b^ ± 7.98	27.81 ^c^ ± 8.14	28.02 ^d^ ± 8.17
	Engagement time (s)	1.20	1.20	1.20	1.20	1.20
VG 46	Max. pressure (bar)	31.49	31.96	32.75	33.38	33.85
	Avg. ± S.D.	24.16 ^ad^ ± 7.11	24.95 ^d^ ± 7.19	25.70 ^e^ ± 7.38	26.19 ^f^ ± 7.52	26.59 ^g^ ± 7.63
	Engagement time (s)	1.20	1.20	1.20	1.20	1.20
VG 68	Max. pressure (bar)	30.87	31.03	31.79	32.40	32.86
	Avg. ± S.D. *	24.37 ^ah^ ± 6.95	25.10 ^h^ ± 6.99	25.58 ^i^ ± 7.18	25.98 ^j^ ± 7.32	25.98 ^kg^ ± 7.43
	Engagement time (s)	1.20	1.20	1.20	1.20	1.20

^a,b,c,d,e,f,g,h,i,j,k^ Means within each column with the same lettering are not significantly different at *p* < 0.05 according to Duncan’s multiple range test; * Avg. ± S.D. is the Average ± Standard Deviation.
